# Glycemic Risk Across Exercise Modalities in Adults with Type 1 Diabetes Using Continuous Glucose Monitoring and Wearable Sensors: A Prospective Cohort Study

**DOI:** 10.3390/jfmk11020231

**Published:** 2026-06-08

**Authors:** Dimna Zoila Alfaro Quezada, Paul César Velásquez Porras, Alicia Olinda Neyra Aranda, Henri Emmanuel López Gómez, Roberto Carlos Dávila-Morán, Vilma Luz Aparicio-Salas, Zoraida Loaiza-Ortiz, Lupe Marilu Huanca Rojas, Digmer Pablo Riquez Livia, Lindomira Castro Llaja, Liliana Inés Romero Núñez

**Affiliations:** 1 Faculty of Health Sciences, Universidad César Vallejo, Lima 15314, Peru; dalfaroq@ucvvirtual.edu.pe (D.Z.A.Q.); pvelasquezp@ucvvirtual.edu.pe (P.C.V.P.); aneyraa@ucvvirtual.edu.pe (A.O.N.A.); 2 Campus Huancayo, Universidad Tecnológica del Perú, Huancayo 12002, Peru; u26214220@utp.edu.pe; 3 Faculty of Social Sciences, Universidad Nacional de San Antonio Abad del Cusco, Cusco 08002, Peru; vilma.aparicio@unsaac.edu.pe; 4 Faculty of Social Communication and Languages, Universidad Nacional de San Antonio Abad del Cusco, Cusco 08002, Peru; zoraida.loaiza@unsaac.edu.pe; 5 Faculty of Education, Universidad Nacional Intercultural de la Selva Central Juan Santos Atahualpa, La Merced 12615, Peru; lhuanca@uniscjsa.edu.pe; 6 Faculty of Education and Physical Culture, Universidad Nacional de Educación Enrique Guzmán y Valle, Lima 15472, Peru; driquez@une.edu.pe; 7 School of Physical Education, Faculty of Health Sciences, Universidad Nacional del Callao, Callao 07011, Peru; lcastrol@unac.edu.pe; 8 Faculty of Business Sciences, Universidad Continental, Huancayo 12001, Peru; lromeron@continental.edu.pe

**Keywords:** type 1 diabetes, exercise modality, glycemic risk, continuous glucose monitoring, wearable sensors, hypoglycemia, real-world cohort, physical activity

## Abstract

**Background**: Exercise provides important health benefits for adults with type 1 diabetes; however, it remains associated with substantial glycemic instability that may vary according to exercise modality, intensity, duration, and clinical context. Continuous glucose monitoring (CGM) and wearable sensors offer an opportunity to characterize exercise-related glycemic responses under real-world conditions, yet prospective free-living data remain limited. **Objective**: This study aimed to evaluate glycemic risk across exercise modalities in adults with type 1 diabetes using CGM and wearable sensors in a real-world prospective cohort. **Methods**: This prospective cohort study was conducted under free-living conditions in 120 adults with type 1 diabetes. Participants were followed during habitual exercise using CGM, wearable sensor data, and session-level exercise classification. A total of 1568 valid exercise sessions were analyzed and categorized as aerobic, resistance, interval-based, or mixed exercise. The primary outcomes were immediate glucose change and time below range during exercise and within 6 h post-exercise. Secondary outcomes included severe biochemical hypoglycemia, time in range, time above range, glycemic variability, delayed hypoglycemia, nocturnal hypoglycemia, and rescue carbohydrate intake. **Results**: Glycemic risk differed across exercise modalities. Aerobic exercise was associated with the greatest immediate glucose decline, the highest time below range, the highest frequency of delayed post-exercise hypoglycemia, and the greatest need for rescue carbohydrate intake. Resistance exercise showed the most favorable acute glycemic profile, whereas interval-based and mixed exercise showed intermediate patterns. The associations between exercise modality and glycemic risk were modified by pre-exercise glucose level, time of day, and insulin delivery modality. Sensitivity analyses were consistent with the primary findings. **Conclusions**: In adults with type 1 diabetes monitored under real-world conditions, glycemic risk varies meaningfully across exercise modalities and is further shaped by clinically relevant contextual factors. These findings support a more individualized interpretation of exercise-related glycemic responses using CGM and wearable-derived data.

## 1. Introduction

Physical activity and structured exercise are central components of long-term care in type 1 diabetes because they support cardiovascular health, metabolic function, physical fitness, and overall well-being. Over the past few years, the evidence base has expanded and recent syntheses have shown that regular physical activity and exercise training can improve glycemic control, body composition, and cardiometabolic risk profiles in people with type 1 diabetes, although the magnitude of benefit has varied across study designs and intervention characteristics [1,2,3]. At the same time, adults with type 1 diabetes often do not achieve recommended activity levels, and both practical and disease-specific barriers continue to limit participation in exercise [4,5]. These observations place exercise at the intersection of therapeutic opportunity and day-to-day clinical complexity in type 1 diabetes.

Exercise nevertheless remains uniquely challenging in type 1 diabetes because exogenous insulin delivery cannot fully reproduce the rapid physiological adjustments that occur during changing energy demands. As a result, acute aerobic activity, resistance exercise, interval-based exercise, and mixed exercise may each produce distinct patterns of glucose decline, hyperglycemia, and glycemic variability before, during, and after exertion [6]. These disturbances are not trivial. They influence confidence, behaviour, carbohydrate intake, insulin dosing, and willingness to engage in regular physical activity, and they remain one of the main reasons why many adults with type 1 diabetes approach exercise with caution [5,6]. The clinical problem is therefore not whether exercise is beneficial, but how to characterize and manage exercise-related glycemic risk with enough precision to support safe participation in real-world settings.

Technological advances have improved this landscape considerably. Continuous glucose monitoring (CGM) has transformed the assessment of glucose dynamics around exercise by enabling near-continuous capture of pre-exercise trajectories, intra-exercise changes, and delayed post-exercise disturbances. Recent standards and evidence syntheses have reinforced the central role of CGM and related diabetes technologies in contemporary type 1 diabetes care, including their ability to improve time in range and reduce clinically important hypoglycemia [7,8]. In parallel, the integration of wearable sensors has broadened the capacity to quantify free-living exercise exposure through heart rate, movement, duration, and intensity proxies, while newer reviews and position statements have clarified how exercise interacts with automated insulin delivery and other technology-enabled strategies [9,10]. However, technological progress has not eliminated uncertainty. Even in the era of CGM, wearables, and automated insulin delivery, exercise continues to provoke substantial glycemic fluctuations, and perceived barriers to physical activity remain common among people living with type 1 diabetes [10,11].

Beyond glucose excursions, ketone-related risk is also relevant around exercise in type 1 diabetes. When pre-exercise hyperglycemia reflects relative insulin deficiency, exercise may worsen ketone accumulation and increase the risk of diabetic ketoacidosis; therefore, ketone assessment remains an important adjunct to glucose monitoring in selected situations.

The literature on modality-specific glycemic responses has also grown, but it has not yet converged on a single risk profile across exercise types. A recent review of postprandial exercise in adults with type 1 diabetes emphasized that the timing and metabolic context of exercise substantially shape glucose responses, with substantial heterogeneity across protocols and individuals [12]. Experimental and pilot work has suggested that acute aerobic exercise often produces larger immediate glucose declines than resistance exercise, whereas resistance-based sessions may attenuate early glucose lowering but do not uniformly prevent later dysglycemia [13]. In the real-world Type 1 Diabetes and Exercise Initiative (T1DEXI), Riddell et al. found that structured aerobic exercise was associated with a greater fall in glucose than resistance exercise, while interval and mixed sessions showed intermediate responses [14]. Subsequent analyses from the same cohort have further shown that higher habitual step counts are generally associated with more favorable overall CGM profiles, but may still coexist with slightly greater exposure to time below range in some activity strata [15], and post hoc CGM analyses of structured aerobic, resistance, interval, and mixed exercise days in the T1DEXI cohort have suggested that exercise can improve composite indices of glycemic status while still generating short-term hypoglycemic vulnerability [16]. More recently, cohort data in young people with type 1 diabetes have indicated that timing and intensity may interact with exercise modality in shaping glycemic outcomes, underscoring that modality alone does not fully explain risk [17].

Important limitations in the current literature help explain these heterogeneous findings. Much of the foundational evidence has come from laboratory-based or tightly standardized protocols, which are essential for mechanistic understanding but offer limited ecological validity when translated to everyday exercise behaviour [6,9,12]. Real-world studies remain comparatively fewer, exercise modality classification has been inconsistent across reports, and many datasets still lack synchronized integration of CGM-derived glucose patterns with wearable-derived activity metrics and contextual variables such as time of day, pre-exercise glucose level, insulin delivery modality, and preceding carbohydrate exposure [9,14,15,16,17]. In addition, several reports have pooled adolescents and adults, mixed structured and unstructured activity, or focused on single-session responses, thereby limiting the ability to estimate differential glycemic risk across exercise modalities specifically in adults with type 1 diabetes followed prospectively under free-living conditions. These gaps are clinically relevant because exercise counselling increasingly depends on individualized interpretation of technology-derived data rather than on generic modality-based rules.

Recent real-world evidence has highlighted both the promise and the remaining limits of current approaches. In the RAPPID study, hypoglycemia and behavioural compensation remained common during free-living unstructured physical activity in adults using automated insulin delivery systems, indicating that advanced insulin delivery does not fully neutralize exercise-related glycemic risk [18]. Data-driven analyses have begun to identify key predictors of exercise-associated dysglycemia, including pre-exercise glucose, prior glucose trends, insulin on board, and exercise type [19,20]. These developments are important, but most predictive work has focused on decision-support tools rather than on prospective, modality-oriented characterization of glycemic risk in adults exercising under real-life conditions. A prospective cohort design that combines CGM with wearable sensor data is therefore well positioned to address a clinically meaningful gap: it can capture repeated exercise exposures in ecologically valid settings, quantify immediate and short-term glycemic responses across modalities, and examine modifiers of risk that are directly actionable in clinical practice.

## 2. Materials and Methods

### 2.1. Study Design and Setting

This quantitative study was conducted as an observational, longitudinal, prospective cohort study under real-world, free-living conditions. A repeated-measures analytical framework was used, with exercise sessions nested within participants. The study was carried out at a single-center outpatient diabetes clinic affiliated with a hospital in Peru, and participant follow-up took place between January 2025 and December 2025. Each participant was followed for 12 weeks from enrollment.

The study was designed to characterize glycemic risk across different exercise modalities during habitual physical activity rather than under laboratory-based exercise protocols. This design was selected to capture ecologically valid exercise exposures and associated glucose responses in everyday settings.

### 2.2. Participants

Adults with type 1 diabetes were recruited by consecutive sampling from outpatient endocrinology and diabetes clinics affiliated with the study center. In addition, potentially eligible individuals receiving routine care at the same center were contacted through a digital invitation system. The target population comprised adults aged 18 to 65 years with a clinical diagnosis of type 1 diabetes for at least 1 year.

Participants were eligible if they had been using real-time CGM for at least 3 months before enrollment, were able to perform habitual physical activity independently, and had regular access to a compatible wearable sensor and smartphone for exercise logging and data synchronization. Participants were excluded if they were pregnant, had end-stage renal disease, had uncontrolled cardiovascular disease that limited exercise participation, were currently enrolled in a supervised exercise trial, were unable to provide informed consent, or had insufficient CGM or wearable data completeness during follow-up. Comorbidities relevant to study eligibility were reviewed during screening to apply the prespecified exclusion criteria; however, additional clinically relevant comorbidities beyond these criteria were not collected in a standardized manner or included as covariates in the present analysis.

At the baseline visit, participants underwent eligibility screening and provided demographic and clinical information. The final provisional sample size was 120 participants. No formal a priori sample size calculation was performed; the sample size was determined pragmatically based on feasibility and the aim of capturing a sufficient number of repeated exercise sessions to support mixed-effects modeling of session-level glycemic outcomes. Analyses were performed at both the participant level and the exercise-session level, with session-level observations serving as the primary repeated unit for the main analyses.

### 2.3. Exercise Modality Classification

The exposure of interest was exercise modality performed during free-living conditions. Exercise sessions were classified into four prespecified categories: aerobic exercise, resistance exercise, interval-based exercise, and mixed exercise.

Aerobic exercise was defined as continuous activity predominantly involving rhythmic whole-body movement and sustained cardiovascular effort, such as brisk walking, jogging, or cycling. Resistance exercise was defined as an activity primarily based on strength-oriented movements using body weight, machines, resistance bands, or external loads. Interval-based exercise was defined as sessions characterized by repeated bouts of higher- and lower-intensity effort within the same session. Mixed exercise was defined as sessions combining aerobic and resistance components within a single exercise bout.

Exercise modality classification was based on participant-reported exercise logs and cross-checked against wearable-derived heart rate and movement patterns. Final session adjudication was performed according to prespecified study rules by two trained researchers. In cases of disagreement, classification was resolved by consensus after joint review of the session record and corresponding wearable data. Sessions with insufficient information to assign a modality category were excluded from session-level analyses.

### 2.4. Data Sources and Measurement Tools

Glucose data were obtained from personal real-time CGM devices (Dexcom G6; Dexcom Inc., San Diego, CA, USA). Device exports were used to obtain interstitial glucose values, time-stamped glucose traces, and derived glucose metrics during the predefined observation windows. Only participants with at least 70% CGM wear time during the observation period were considered eligible for analysis.

Physical activity data were obtained from a wrist-worn multisensor wearable device (Polar Ignite 3, Polar Electro Oy, Kempele, Finland) that recorded exercise timing, heart rate, duration, and activity-related metrics. Wearable data were used to verify exercise-session timing and to support modality classification. For a session to be considered valid for exercise-session-level analysis, wearable data had to provide valid heart rate and timing data for at least 80% of the recorded session duration.

Contextual exercise information was collected using a study-specific smartphone-based digital exercise log imple-mented in Google Forms (Google LLC, Mountain View, CA, USA), completed as soon as possible after each exercise session. The log could also capture exercise-related carbohydrate intake and rescue carbohydrate intake, but these carbohydrate-related entries were not uniformly available for all sessions. Exercise-related carbohydrate intake referred to carbohydrate consumed before, during, or shortly after exercise in temporal relation to the session as part of planned or contextual peri-exercise management. Rescue carbohydrate intake referred to rapid-acting carbohydrate consumed during exercise or within the early post-exercise window in response to symptoms, CGM trends, measured hypoglycemia, or perceived risk of impending hypoglycemia. Because these fields were incompletely captured, carbohydrate-related variables were treated as exploratory contextual measures. Missing entries were handled as missing data and were not imputed.

### 2.5. Outcome Measures

The primary outcome was glycemic risk across exercise modalities. The main primary endpoint was time below range (TBR; glucose < 70 mg/dL) during exercise and within the 6-h post-exercise period. A co-primary acute endpoint was immediate glucose change, defined as the difference between glucose measured 30 min before exercise onset and glucose measured at the end of the exercise session.

Secondary glycemic outcomes included TBR < 54 mg/dL, time in range (TIR; 70–180 mg/dL), time above range (TAR; >180 mg/dL), TAR > 250 mg/dL, mean glucose during exercise and in the post-exercise period, and glycemic variability indices, including glucose standard deviation and coefficient of variation. Additional secondary outcomes included delayed post-exercise hypoglycemia within 6 h after exercise, nocturnal hypoglycemia during the subsequent night, and rescue carbohydrate intake. Analyses of rescue carbohydrate intake were based on sessions with available rescue carbohydrate entries and were interpreted as exploratory because these data were not captured with the same level of standardization as CGM and wearable-derived variables.

The observation windows were defined a priori. The pre-exercise window comprised the 30 min before exercise onset. The intra-exercise window extended from exercise onset to exercise end. The early post-exercise window included the 6 h immediately following exercise completion. The nocturnal window was defined as 00:00 to 06:00 h after an exercise session performed on the same calendar day.

### 2.6. Data Collection and Session Verification

All participants attended a baseline visit at enrollment. At that visit, eligibility was confirmed, and baseline demographic and clinical data were recorded. Participants were then instructed to continue their usual exercise habits under free-living conditions throughout follow-up. No experimental exercise intervention was imposed.

CGM data were exported from Dexcom Clarity (Dexcom Inc., San Diego, CA, USA), and wearable data were ex-ported from Polar Flow (Polar Electro Oy, Kempele, Finland). Data were synchronized weekly through secure digi-tal export procedures. Exercise sessions were first identified using the participant-completed digital logs and were then verified against wearable-derived timestamps and physiological activity signals. When more than one exercise session occurred on the same day, each session was treated as a separate observation if distinct timing and modality could be established.

Session-level records were linked to CGM data using time-matched synchronization procedures. Records were checked for temporal consistency between exercise logs, wearable timestamps, and CGM traces. Sessions were excluded from exercise-session-level analyses if they had incomplete timing data, missing modality classification, invalid CGM-wearable synchronization, or insufficient signal quality for outcome derivation. Implausible records and duplicate entries were reviewed manually before exclusion. Data were stored in pseudonymized form on password-protected institutional servers accessible only to authorized study personnel.

### 2.7. Statistical Analysis

Continuous variables were summarized as mean ± standard deviation or median and interquartile range, depending on distributional characteristics. Categorical variables were summarized as counts and percentages. Normality was assessed using the Shapiro–Wilk test and visual inspection of histograms and Q–Q plots. The primary analyses were performed at the exercise-session level using mixed-effects regression models to account for repeated sessions nested within participants. A participant-specific random intercept was included to model within-subject correlation. Exercise modality was entered as the primary fixed-effect exposure. Pre-exercise glucose level, time of day, insulin delivery modality, age, sex, diabetes duration, and session duration were included as covariates in adjusted models. Interaction terms of interest included exercise modality × pre-exercise glucose, exercise modality × time of day, and exercise modality × insulin delivery modality. Linear mixed-effects models were used for continuous outcomes, including immediate glucose change, TBR percentage, TAR percentage, TIR percentage, and glycemic variability indices. Mixed-effects logistic regression models were used for binary outcomes, such as the occurrence of hypoglycemia during or after exercise. Sensitivity analyses were performed by excluding sessions with extreme pre-exercise hyperglycemia (defined a priori as pre-exercise glucose > 250 mg/dL), excluding sessions with missing covariate data for the adjusted primary model, and stratifying analyses according to insulin delivery modality. A two-sided *p*-value < 0.05 was considered statistically significant. For secondary outcomes, false discovery rate correction was applied. Missing data were handled by complete-case analysis in the primary models. Multiple imputation was performed as a sensitivity approach when the pattern and extent of missingness supported such analyses. All statistical analyses were performed using R version 4.3.2 (R Foundation for Statistical Computing, Vienna, Austria). Analytical code was available from the corresponding author upon reasonable request.

### 2.8. Ethical Considerations

The study was conducted in accordance with the Declaration of Helsinki and approved by the Institutional Review Board of Universidad Nacional de Educación Enrique Guzmán y Valle (approval code: UNE-REC-2024-016; date of approval: 16 December 2024). All participants provided written informed consent before enrollment. Participant confidentiality was protected through pseudonymization of exported CGM and wearable data and storage on secure institutional servers.

## 3. Results

### 3.1. Participant and Exercise-Session Characteristics

A total of 148 individuals were screened for eligibility, of whom 128 were enrolled. Eight participants were excluded after enrollment because CGM completeness was below the prespecified threshold (*n* = 5) or because wearable data were incomplete (*n* = 3). The final analytical sample, therefore, comprised 120 adults with type 1 diabetes.

Mean age was 34.8 ± 10.2 years, mean diabetes duration was 15.1 ± 8.7 years, mean body mass index was 25.3 ± 3.8 kg/m^2^, and mean baseline HbA1c was 7.5 ± 0.8% (58.5 ± 8.7 mmol/mol). Overall, 62 participants (51.7%) were female. Regarding insulin delivery modality, 41 participants (34.2%) used multiple daily injections, 37 (30.8%) used insulin pump therapy, and 42 (35.0%) used automated insulin delivery (Table 1).

During follow-up, 1742 exercise sessions were recorded. Of these, 1568 met the prespecified criteria for valid session-level analysis, whereas 174 sessions were excluded because of incomplete timing data, missing modality classification, or invalid CGM–wearable synchronization. The median number of valid exercise sessions per participant was 12 (interquartile range, 9–16). Same-day repeated exercise sessions occurred on 86 participant-days and accounted for 178 valid sessions. These sessions were retained as separate observations when distinct timing and modality could be established.

### 3.2. Distribution of Exercise Modalities

Among the 1568 valid exercise sessions, 692 (44.1%) were classified as aerobic exercise, 348 (22.2%) as resistance exercise, 236 (15.1%) as interval-based exercise, and 292 (18.6%) as mixed exercise (Table 2). Aerobic exercise was therefore the most frequently recorded modality.

Mean session duration differed across modality categories, ranging from 31.4 ± 10.9 min for interval-based exercise to 47.2 ± 15.8 min for aerobic exercise. Wearable-derived mean heart rate was highest during interval-based sessions (146 ± 19 bpm) and lowest during resistance sessions (118 ± 16 bpm). Pre-exercise glucose levels were broadly comparable across modalities.

With respect to time of day, 436 sessions (27.8%) were performed in the morning, 701 (44.7%) in the afternoon, and 431 (27.5%) in the evening. The proportion of evening sessions was highest for aerobic exercise.

### 3.3. Exercise Modality Differences in Immediate Glucose Change and Time Below Range

The primary endpoint, time below range (TBR < 70 mg/dL) during exercise and within the 6 h post-exercise window, differed across exercise modalities (overall *p* < 0.001). In unadjusted session-level analyses, mean TBR was 5.8 ± 4.7% for aerobic exercise, 2.6 ± 3.1% for resistance exercise, 4.1 ± 4.0% for interval-based exercise, and 3.8 ± 3.8% for mixed exercise (Table 3).

In adjusted mixed-effects models, aerobic exercise was associated with significantly higher TBR than resistance exercise (β = +2.91 percentage points, 95% CI 2.10 to 3.72, *p* < 0.001). Interval-based exercise also showed higher TBR than resistance exercise (β = +1.24 percentage points, 95% CI 0.35 to 2.13, *p* = 0.006), as did mixed exercise (β = +0.96 percentage points, 95% CI 0.12 to 1.80, *p* = 0.025).

The co-primary acute endpoint, immediate glucose change from 30 min before exercise onset to the end of the session, also differed across modalities (overall *p* < 0.001). Aerobic exercise was associated with the greatest glucose decline (−38.4 ± 23.5 mg/dL), whereas resistance exercise showed the smallest decline (−11.2 ± 19.4 mg/dL). Interval-based exercise (−24.6 ± 21.7 mg/dL) and mixed exercise (−21.3 ± 20.8 mg/dL) showed intermediate responses.

After adjustment, aerobic exercise remained associated with a larger acute glucose decline than resistance exercise (β = −26.8 mg/dL, 95% CI −31.5 to −22.1, *p* < 0.001). Interval-based exercise (β = −13.7 mg/dL, 95% CI −18.9 to −8.5, *p* < 0.001) and mixed exercise (β = −10.1 mg/dL, 95% CI −15.0 to −5.2, *p* < 0.001) also showed significantly greater glucose decline than resistance exercise.

Taken together, the primary analyses indicated that glycemic risk was not uniform across exercise modalities and that aerobic exercise showed the least favorable acute glycemic profile among the four modality categories evaluated.

### 3.4. Secondary Glycemic Outcomes

Secondary CGM-derived outcomes are summarized in Table 4. TBR < 54 mg/dL differed across modalities (overall *p* < 0.001), occurring in 112 aerobic sessions (16.2%), 26 resistance sessions (7.5%), 32 interval-based sessions (13.6%), and 33 mixed sessions (11.3%).

Time in range (70–180 mg/dL) also differed across modalities (overall *p* < 0.001). Mean TIR was highest during resistance exercise (75.4 ± 12.8%) and lowest during aerobic exercise (68.9 ± 14.6%). Time above range > 180 mg/dL showed smaller but statistically significant differences across modalities (overall *p* = 0.018), with resistance exercise showing the highest post-exercise TAR > 180 mg/dL.

Glycemic variability, expressed as the coefficient of variation, differed across modalities (overall *p* < 0.001). The highest variability was observed during aerobic sessions (34.1 ± 8.2%), whereas the lowest was observed during resistance sessions (29.8 ± 7.4%).

Delayed post-exercise hypoglycemia within 6 h occurred in 147 aerobic sessions (21.2%), 39 resistance sessions (11.2%), 41 interval-based sessions (17.4%), and 48 mixed sessions (16.4%) (overall *p* < 0.001). Nocturnal hypoglycemia during the subsequent night occurred after 54 aerobic sessions (7.8%), 15 resistance sessions (4.3%), 13 interval-based sessions (5.5%), and 18 mixed sessions (6.2%), with no statistically significant overall difference across modalities (*p* = 0.061).

Among sessions with available rescue carbohydrate entries (*n* = 1259), rescue carbohydrate intake was reported in 249 sessions (19.8%) overall and was most frequent during or after aerobic exercise (25.6%), followed by interval-based exercise (18.6%), mixed exercise (17.1%), and resistance exercise (11.2%) (overall *p* < 0.001).

### 3.5. Interaction and Sensitivity Analyses

A significant interaction was observed between exercise modality and pre-exercise glucose level (interaction *p* = 0.002). The adjusted difference in TBR between aerobic and resistance exercise was greatest when pre-exercise glucose was <140 mg/dL (β = +3.62 percentage points, 95% CI 2.54 to 4.70, *p* < 0.001) and smaller when pre-exercise glucose was ≥180 mg/dL (β = +1.18 percentage points, 95% CI 0.22 to 2.14, *p* = 0.016) (Table 5).

The interaction between exercise modality and time of day was also significant (interaction *p* = 0.031). The aerobic-versus-resistance difference in TBR increased progressively from morning to evening, with the largest estimate observed in evening sessions (β = +3.44 percentage points, 95% CI 2.29 to 4.59, *p* < 0.001).

A significant interaction was identified between exercise modality and insulin delivery modality (interaction *p* = 0.044). In stratified analyses, the aerobic-versus-resistance difference in TBR was largest in participants using multiple daily injections (β = +3.51 percentage points, 95% CI 2.33 to 4.69, *p* < 0.001), intermediate in pump users (β = +2.76 percentage points, 95% CI 1.59 to 3.93, *p* < 0.001), and attenuated in automated insulin delivery users (β = +1.94 percentage points, 95% CI 0.82 to 3.06, *p* = 0.001).

Sensitivity analyses were consistent with the primary findings. After exclusion of 42 sessions with pre-exercise glucose > 250 mg/dL (1526 sessions retained), the adjusted aerobic-versus-resistance difference in TBR remained significant (β = +2.67 percentage points, 95% CI 1.88 to 3.46, *p* < 0.001). Analyses restricted to sessions with complete contextual covariate data for the adjusted primary model (1496 sessions; 72 excluded because of missing covariates) yielded comparable results (β = +2.83 percentage points, 95% CI 2.01 to 3.65, *p* < 0.001).

## 4. Discussion

This prospective cohort study provides real-world evidence that glycemic risk differs meaningfully across exercise modalities in adults with type 1 diabetes using continuous glucose monitoring and wearable sensors. The overall pattern was consistent across the main analyses: aerobic exercise was associated with the greatest immediate glucose decline, the highest exposure to time below range, and the greatest short-term need for rescue carbohydrate intake, whereas resistance exercise showed the most favorable acute glycemic profile. Interval-based and mixed exercise occupied an intermediate position between these two extremes. The results also indicate that modality-specific risk should not be understood as fixed, because the association between exercise modality and glycemic outcomes was modified by pre-exercise glucose level, time of day, and insulin delivery modality. In this respect, the present findings support the first two working hypotheses and refine the third by showing that contextual factors remain clinically relevant even when exercise is monitored with modern diabetes technology.

The first working hypothesis was therefore supported. In the present cohort, aerobic exercise was associated with greater immediate glucose decline and higher time below range than resistance exercise. This interpretation is physiologically coherent and aligns with the current understanding that different exercise modalities impose distinct balances between peripheral glucose disposal, circulating insulin action, and counterregulatory hormone responses in type 1 diabetes [6,12]. The second hypothesis was also supported, as interval-based and mixed exercise showed intermediate glycemic profiles rather than behaving like purely aerobic or purely resistance exercise. This intermediate pattern is clinically important because it suggests that exercise modality cannot be reduced to a simple binary distinction; instead, glycemic risk appears to follow a graded continuum shaped by the metabolic structure of the activity itself. The third hypothesis was likewise supported. The significant interactions observed for pre-exercise glucose level, time of day, and insulin delivery modality indicate that modality-specific glycemic risk is conditional rather than uniform, and that the same exercise category may carry substantially different short-term glycemic consequences depending on the surrounding clinical context.

These findings are broadly consistent with the recent evidence base on exercise-related glycemic responses in type 1 diabetes. The most direct point of comparison is the T1DEXI, which showed in a real-world at-home setting that aerobic exercise produced a larger glucose decline than interval or resistance exercise, regardless of insulin delivery modality [14]. Our findings extend that general pattern by suggesting that, under free-living conditions and across repeated naturally occurring exercise sessions, the same hierarchy remains evident not only for immediate glucose change but also for short-term exposure to hypoglycemia-related metrics. This is important because it indicates that the modality-specific signal described in structured real-world exercise sessions persists in a more ecologically complex setting. The present results are also compatible with the 2023 review by Helleputte et al., which emphasized that several forms of postprandial exercise in adults with type 1 diabetes tend to lower glucose concentration, while the magnitude and safety of that decline depend strongly on timing, intensity, and accompanying insulin adjustments [12]. More broadly, the current study fits with the wider literature showing that regular physical activity remains beneficial overall in type 1 diabetes, even though short-term glycemic instability continues to complicate exercise participation [6,10].

The intermediate profile observed for interval-based and mixed exercise also deserves emphasis. Prior work has increasingly suggested that exercise modalities incorporating intermittent higher-intensity efforts or resistance components may attenuate immediate glucose decline relative to continuous aerobic exercise, although they do not eliminate glycemic disturbances altogether [1,6,9]. The present findings are in line with that view. They suggest that mixed and interval-based exercise may occupy a clinically relevant middle ground, with less acute glucose lowering than aerobic exercise but more variability than resistance exercise alone. This interpretation also resonates with recent analyses from T1DEXI showing that exercise can improve broader composite glycemic metrics despite a modest increase in time below range, as well as with step-count analyses suggesting that more activity may improve overall CGM profiles while still modestly increasing hypoglycemia exposure in some circumstances [15,16]. In parallel, Jacobs et al. have shown that real-world adherence to exercise position-statement strategies in T1DEXI is variable, which reinforces the likelihood that modality-related effects in everyday life are being shaped by heterogeneous behavioral adjustments rather than by exercise physiology alone [21].

The observed effect modification by pre-exercise glucose level, time of day, and insulin delivery modality is particularly relevant for interpretation. The stronger aerobic-versus-resistance difference at lower pre-exercise glucose levels is consistent with earlier guidance that starting glycemia is a key determinant of both the direction and magnitude of exercise-related glucose excursions [9,22]. Likewise, the greater apparent risk in evening sessions is compatible with prior literature indicating that the timing of exercise matters, especially because late-day activity may interact with residual insulin action, post-exercise recovery, and vulnerability to delayed or nocturnal hypoglycemia [12,17,22]. The attenuation of modality-related risk in automated insulin delivery users is also plausible in light of emerging data showing that AID systems can reduce, but not abolish, exercise-related glycemic instability [10,18,23,24]. The recent EASD/ISPAD position statement has emphasized that exercise still challenges current AID systems despite their clear benefits, while the RAPPID study showed that unstructured physical activity continues to provoke hypoglycemia and compensatory behavioral adjustments even among adults using contemporary automated insulin delivery systems [10,18]. Our findings fit that perspective well: technology appears to mitigate risk, but it does not remove the importance of exercise modality or the need for contextual interpretation.

From a clinical and scientific perspective, these findings support a more individualized approach to exercise counseling in adults with type 1 diabetes. Recent reviews and position statements have increasingly argued that exercise guidance should move beyond generic advice and instead incorporate modality, timing, device use, and surrounding behavioral context [9,10,22]. The present study reinforces that position. In practical terms, the data suggest that an aerobic session performed at lower starting glucose and later in the day should be interpreted differently from a resistance session undertaken at higher starting glucose or under automated insulin delivery. This has implications for how clinicians and patients use CGM trend data, wearable-derived activity signals, and device context when preparing for, monitoring, and reviewing exercise. It also matters for patient confidence. Fear of hypoglycemia remains one of the most persistent barriers to physical activity in type 1 diabetes, even in the era of advanced glucose-sensing technologies [5,11]. Recent registry and survey data indicate that many physically active adults continue to rely heavily on trial-and-error approaches despite widespread technology use [5,11]. Accordingly, the present findings may be most useful not as rigid exercise rules, but as evidence to inform more precise, scenario-based exercise management and education.

These findings also have practical nutritional implications. Before exercise, individualized carbohydrate planning and insulin adjustment remain central, particularly when starting glucose is in the lower range or when recent prandial insulin is still active; when glucose is unexpectedly high, ketone assessment is more informative than additional carbohydrate intake. During prolonged or predominantly aerobic sessions, rapid-acting carbohydrate supplementation guided by CGM trends, session duration, and symptoms may help attenuate excessive glucose decline. After exercise, recovery strategies should prioritize individualized carbohydrate replacement and insulin review to reduce delayed hypoglycemia, while emerging evidence suggests that post-exercise protein intake may also help lessen late glucose decline in some settings, although this evidence remains preliminary and should be interpreted cautiously. A concise literature-informed summary of these practical considerations is provided in Table S1.

This study has several strengths. Its prospective cohort design, free-living setting, and repeated session-level observations nested within individuals allowed the analysis to reflect exercise as it occurs in daily life rather than under tightly controlled laboratory conditions. The combined use of continuous glucose monitoring and wearable sensor data improved temporal alignment between exercise exposure and glycemic response and permitted the examination of clinically relevant modifiers, including pre-exercise glucose level, time of day, and insulin delivery modality. At the same time, several limitations should be considered. First, the observational design does not support causal inference and cannot exclude residual confounding. Second, exercise modality classification may have been imperfect, especially for mixed or poorly documented sessions. Third, contextual behavioral data were incompletely characterized: exercise-related carbohydrate intake and rescue carbohydrate intake were not captured uniformly across all sessions or with the same level of standardization as CGM and wearable variables, and meal timing, meal composition, insulin dose adjustments, and rating of perceived exertion were not systematically collected or were not available at sufficient completeness for formal analysis. Fourth, exercise intensity was inferred primarily from wearable-derived proxies rather than from laboratory-based physiological measurements; therefore, sessions within the same broad modality may still have differed meaningfully in metabolic stress across individuals. Fifth, when more than one exercise session occurred on the same day, later glycemic responses may have been influenced by residual effects of prior activity, altered insulin sensitivity, carbohydrate replacement, or accumulated fatigue. Sixth, other clinically relevant comorbidities beyond the prespecified exclusion criteria were not systematically characterized or analyzed in the present study. Finally, the single-center design and the inclusion of adults already using CGM and wearable devices may limit generalizability to broader populations with type 1 diabetes, particularly those with lower technology engagement or different exercise behaviors. An additional limitation is that we did not measure blood or urine ketones. Accordingly, while the present study characterizes glycemic instability around exercise, it cannot quantify ketosis-related risk, particularly in sessions beginning with marked hyperglycemia or relative insulin deficiency. Taken together, these limitations reinforce that exercise-related glycemic responses should be interpreted within their broader behavioral and clinical context, even when CGM and wearable technologies are available.

Several future directions follow directly from these findings. Multicenter real-world cohorts with broader demographic, behavioral, and technological diversity are needed to determine whether the present modality-specific patterns remain stable across different care settings and patient profiles. More granular integration of insulin dosing, meal timing, carbohydrate intake, and AID algorithm data would help clarify how much of the observed modality effect is attributable to exercise physiology itself versus peri-exercise management decisions [10,18,24]. Adjunctive pharmacotherapies may also alter the metabolic context in which exercise is undertaken; for example, emerging studies in adults with type 1 diabetes and overweight or obesity suggest that semaglutide may reduce body weight and insulin requirements in selected patients, although this therapy was not evaluated in our cohort and remains outside the main scope of the present analysis [25,26]. Future real-world studies should integrate ketone monitoring with CGM and wearable-derived exercise data to provide a broader safety profile around physical activity in adults with type 1 diabetes. There is also a need to validate wearable-based approaches for classifying exercise modality more rigorously, especially in mixed and spontaneous free-living activity. In parallel, the field is now well positioned to move toward pragmatic intervention studies and hybrid observational-interventional designs that test modality-specific decision support strategies in everyday care. Finally, the development of predictive tools that combine exercise modality, CGM trends, and contextual device data for real-time risk stratification represents a particularly promising next step, as recent modeling work has already shown that exercise-related glycemic events can be forecast with clinically useful performance using technology-derived data streams [20].

Additional mechanistic context may also help interpret the comparatively favorable acute glycemic profile observed with resistance exercise in the present cohort. Controlled human studies have shown that resistance exercise produces smaller immediate glucose declines than aerobic exercise in adults with type 1 diabetes, and that performing resistance exercise before aerobic exercise can improve glycemic stability across the exercise bout [27,28]. This pattern is consistent with physiological literature suggesting that resistance exercise relies more heavily on non-oxidative and glycolytic muscle metabolism than continuous aerobic exercise, potentially providing transient protection against early glucose decline through counterregulatory hormonal responses and short-term post-exercise glucose preservation, although delayed dysglycemia may still occur [29]. In parallel, skeletal-muscle–targeted translational work has identified myostatin as a pathway of interest in type 1 diabetes: serum myostatin appears elevated in humans with type 1 diabetes, while genetic or pharmacologic myostatin inhibition in insulin-deficient models has preserved muscle mass and aspects of metabolic or musculoskeletal function [30,31,32,33]. These mechanisms were not directly measured in our cohort and should therefore be interpreted as biologically plausible context rather than causal explanations of the present real-world associations.

In summary, the present study adds real-world evidence that glycemic risk varies across exercise modalities in adults with type 1 diabetes and that this variation is meaningfully shaped by clinically actionable contextual factors. Aerobic exercise appears to carry the greatest acute and short-term hypoglycemia-related burden, resistance exercise the lowest, and interval-based and mixed exercise intermediate profiles. The broader implication is not that one modality is universally “safe” or “unsafe”, but that exercise-related glycemic risk is better understood as a dynamic interaction between modality, starting glycemia, timing, and insulin delivery context. That perspective is consistent with the direction in which the field has been moving: toward more precise, technology-enabled, and individualized exercise guidance for people living with type 1 diabetes.

## 5. Conclusions

In this prospective real-world cohort of adults with type 1 diabetes, glycemic risk differed meaningfully across exercise modalities, with aerobic exercise showing the least favorable acute glycemic profile, resistance exercise the most favorable profile, and mixed and interval-based exercise intermediate patterns. The study therefore addressed its main objective and supported the working hypotheses that exercise modality is an important determinant of short-term glycemic response and that this relationship is modified by clinically relevant contextual factors, including pre-exercise glucose level, time of day, and insulin delivery modality. By combining continuous glucose monitoring and wearable sensor data under free-living conditions, this study adds clinically relevant evidence beyond tightly controlled laboratory settings and supports a more individualized interpretation of exercise-related glycemic risk in adults with type 1 diabetes. These conclusions should be interpreted in light of the observational design and the real-world variability inherent to exercise behavior, but they also highlight the value of future studies integrating more granular insulin, meal, and device data to further refine modality-specific risk stratification and exercise guidance.

## Figures and Tables

**Table 1:** Baseline demographic and clinical characteristics of the study participants.

Variable	Value
Age, years	34.8 ± 10.2
Female sex, *n* (%)	62 (51.7)
Diabetes duration, years	15.1 ± 8.7
Body mass index, kg/m^2^	25.3 ± 3.8
HbA1c, %	7.5 ± 0.8
HbA1c, mmol/mol	58.5 ± 8.7
Multiple daily injections, *n* (%)	41 (34.2)
Insulin pump therapy, *n* (%)	37 (30.8)
Automated insulin delivery, *n* (%)	42 (35.0)

**Table 2:** Exercise-session characteristics according to exercise modality.

Characteristic	Aerobic (*n* = 692)	Resistance (*n* = 348)	Interval-Based (*n* = 236)	Mixed (*n* = 292)
Sessions, *n* (%)	692 (44.1)	348 (22.2)	236 (15.1)	292 (18.6)
Duration, min	47.2 ± 15.8	41.6 ± 13.9	31.4 ± 10.9	38.7 ± 12.8
Mean heart rate, bpm	132 ± 18	118 ± 16	146 ± 19	136 ± 17
Pre-exercise glucose, mg/dL	162 ± 34	158 ± 32	160 ± 33	159 ± 31
Morning sessions, *n* (%)	172 (24.9)	114 (32.8)	58 (24.6)	92 (31.5)
Afternoon sessions, *n* (%)	304 (43.9)	157 (45.1)	111 (47.0)	129 (44.2)
Evening sessions, *n* (%)	216 (31.2)	77 (22.1)	67 (28.4)	71 (24.3)

**Table 3:** Primary glycemic outcomes across exercise modalities.

Modality	Immediate Glucose Change, mg/dL (Raw)	TBR < 70 mg/dL During Exercise + 6 h Post, % (Raw)	Adjusted Difference in Immediate Glucose Change vs. Resistance (95% CI)	*p*-Value	Adjusted Difference in TBR vs. Resistance, Percentage Points (95% CI)	*p*-Value
Resistance	−11.2 ± 19.4	2.6 ± 3.1	Reference	—	Reference	—
Aerobic	−38.4 ± 23.5	5.8 ± 4.7	−26.8 (−31.5 to −22.1)	<0.001	+2.91 (2.10 to 3.72)	<0.001
Interval-based	−24.6 ± 21.7	4.1 ± 4.0	−13.7 (−18.9 to −8.5)	<0.001	+1.24 (0.35 to 2.13)	0.006
Mixed	−21.3 ± 20.8	3.8 ± 3.8	−10.1 (−15.0 to −5.2)	<0.001	+0.96 (0.12 to 1.80)	0.025

**Table 4:** Secondary glycemic outcomes according to exercise modalities.

Outcome	Aerobic (*n* = 692)	Resistance (*n* = 348)	Interval-Based (*n* = 236)	Mixed (*n* = 292)	Overall *p*-Value
TBR < 54 mg/dL, *n* (%)	112 (16.2)	26 (7.5)	32 (13.6)	33 (11.3)	<0.001
TIR 70–180 mg/dL, %	68.9 ± 14.6	75.4 ± 12.8	71.6 ± 13.7	72.5 ± 13.2	<0.001
TAR > 180 mg/dL, %	18.5 ± 12.2	21.8 ± 13.1	19.6 ± 12.8	20.2 ± 12.7	0.018
Coefficient of variation, %	34.1 ± 8.2	29.8 ± 7.4	32.5 ± 7.9	31.9 ± 7.6	<0.001
Delayed hypoglycemia within 6 h, *n* (%)	147 (21.2)	39 (11.2)	41 (17.4)	48 (16.4)	<0.001
Nocturnal hypoglycemia, *n* (%)	54 (7.8)	15 (4.3)	13 (5.5)	18 (6.2)	0.061
Rescue carbohydrate intake among sessions with available entries, *n*/N (%)	142/555 (25.6)	31/276 (11.2)	36/194 (18.6)	40/234 (17.1)	<0.001

Note: Rescue carbohydrate intake was analyzed among sessions with available rescue carbohydrate entries. Missing entries were treated as missing data and were not imputed. The denominators for this exploratory contextual variable differ from the total number of valid exercise sessions because rescue carbohydrate entries were not uniformly available across all sessions.

**Table 5:** Interaction and sensitivity analyses for the primary outcome (TBR < 70 mg/dL).

Analysis	Estimate for Aerobic vs. Resistance (95% CI)	*p*-Value
Interaction by pre-exercise glucose		
<140 mg/dL	+3.62 percentage points (2.54 to 4.70)	<0.001
140–179 mg/dL	+2.41 percentage points (1.46 to 3.36)	<0.001
≥180 mg/dL	+1.18 percentage points (0.22 to 2.14)	0.016
Interaction *p*-value		0.002
Interaction by time of day		
Morning	+2.05 percentage points (1.08 to 3.02)	<0.001
Afternoon	+2.88 percentage points (1.91 to 3.85)	<0.001
Evening	+3.44 percentage points (2.29 to 4.59)	<0.001
Interaction *p*-value		0.031
Interaction by insulin delivery modality		
Multiple daily injections	+3.51 percentage points (2.33 to 4.69)	<0.001
Insulin pump therapy	+2.76 percentage points (1.59 to 3.93)	<0.001
Automated insulin delivery	+1.94 percentage points (0.82 to 3.06)	0.001
Interaction *p*-value		0.044
Sensitivity analyses		
Excluding sessions with pre-exercise glucose > 250 mg/dL (excluded *n* = 42; retained *n* = 1526)	+2.67 percentage points (1.88 to 3.46)	<0.001
Restricting to sessions with complete contextual covariates (excluded *n* = 72; retained *n* = 1496)	+2.83 percentage points (2.01 to 3.65)	<0.001

## Data Availability

The data that support the findings of this study are available from the corresponding author upon reasonable request. The dataset contains information that could compromise the privacy of study participants and is therefore not publicly shared. Access will be granted to qualified researchers who present an ethically approved protocol and agree to a data-use agreement.

## Supplementary Materials

The following supporting information can be downloaded at: https://www.mdpi.com/article/10.3390/jfmk11020231/s1, Table S1: Practical nutrition considerations before, during, and after exercise in adults with type 1 diabetes. References [6,10,12,22,34,35] are cited in the Supplementary Materials.

## References

[1] De Cock D., Schreurs L., Steenackers N., Pazmino S., Cools W., Eykerman L., Thiels H., Mathieu C., Van der Schueren B. (2024). The Effect of Physical Activity on Glycaemic Control in People with Type 1 Diabetes Mellitus: A Systematic Literature Review and Meta-analysis. Diabet. Med..

[2] Khalafi M., Dinizadeh F., Rosenkranz S.K., Symonds M.E., Fatolahi S. (2025). The Effects of Exercise Training on Body Composition and Cardiometabolic Risk Factors in Type 1 Diabetes Mellitus: A Systematic Review and Meta-Analysis. Healthcare.

[3] Alvares T.S., De Souza L.V.M., Soares R.N., Lessard S.J. (2024). Cardiorespiratory Fitness Is Impaired in Type 1 and Type 2 Diabetes: A Systematic Review, Meta-Analysis, and Meta-Regression. Med. Sci. Sports Exerc..

[4] Johansen R.F., Caunt S., Heller S., Sander S.E., Søndergaard E., Molsted S., Kristensen P.L. (2024). Factors Influencing Physical Activity Level in Adults with Type 1 Diabetes: A Cross-Sectional Study. Can. J. Diabetes.

[5] Alobaid A.M., Zulyniak M.A., Ajjan R.A., Brož J., Hopkins M., Campbell M.D. (2023). Barriers to Exercise in Adults with Type 1 Diabetes and Insulin Resistance. Can. J. Diabetes.

[6] Riddell M.C., Peters A.L. (2023). Exercise in Adults with Type 1 Diabetes Mellitus. Nat. Rev. Endocrinol..

[7] ElSayed N.A., McCoy R.G., Aleppo G., Balapattabi K., Beverly E.A., Briggs Early K., Bruemmer D., Echouffo-Tcheugui J.B., Ekhlaspour L., Garg R. (2025). 7. Diabetes Technology: Standards of Care in Diabetes—2025. Diabetes Care.

[8] Rizos E.C., Markozannes G., Charitakis N., Filis P., Stoimeni A.E., Nørgaard K., Ntzani E.E., Tsilidis K.K. (2025). Continuous Glucose Monitoring in Type 1 Diabetes, Type 2 Diabetes, and Diabetes During Pregnancy: A Systematic Review with Meta-Analysis of Randomized Controlled Trials. Diabetes Technol. Ther..

[9] Perkins B.A., Turner L.V., Riddell M.C. (2024). Applying Technologies to Simplify Strategies for Exercise in Type 1 Diabetes. Diabetologia.

[10] Moser O., Zaharieva D.P., Adolfsson P., Battelino T., Bracken R.M., Buckingham B.A., Danne T., Davis E.A., Dovč K., Forlenza G.P. (2025). The Use of Automated Insulin Delivery around Physical Activity and Exercise in Type 1 Diabetes: A Position Statement of the European Association for the Study of Diabetes (EASD) and the International Society for Pediatric and Adolescent Diabetes (ISPAD). Diabetologia.

[11] Guédet C., Alexandre-Heymann L., Yardley J.E., Messier V., Boudreau V., Chahal T., Dostie M., Mathieu M.-E., Brazeau A.-S., Tagougui S. (2025). Exploring Perceived Barriers to Physical Activity among Individuals with Type 1 Diabetes in the Era of New Technologies: An Analysis from the BETTER Registry. Diabetes Metab..

[12] Helleputte S., Yardley J.E., Scott S.N., Stautemas J., Jansseune L., Marlier J., De Backer T., Lapauw B., Calders P. (2023). Effects of Postprandial Exercise on Blood Glucose Levels in Adults with Type 1 Diabetes: A Review. Diabetologia.

[13] Ivandic M., Cigrovski Berkovic M., Ormanac K., Sabo D., Omanovic Kolaric T., Kuna L., Mihaljevic V., Canecki Varzic S., Smolic M., Bilic-Curcic I. (2023). Management of Glycemia during Acute Aerobic and Resistance Training in Patients with Diabetes Type 1: A Croatian Pilot Study. Int. J. Environ. Res. Public Health.

[14] Riddell M.C., Li Z., Gal R.L., Calhoun P., Jacobs P.G., Clements M.A., Martin C.K., Doyle Iii F.J., Patton S.R., Castle J.R. (2023). Examining the Acute Glycemic Effects of Different Types of Structured Exercise Sessions in Type 1 Diabetes in a Real-World Setting: The Type 1 Diabetes and Exercise Initiative (T1DEXI). Diabetes Care.

[15] Turner L.V., Marak M.C., Gal R.L., Calhoun P., Li Z., Jacobs P.G., Clements M.A., Martin C.K., Doyle F.J., Patton S.R. (2024). Associations between Daily Step Count Classifications and Continuous Glucose Monitoring Metrics in Adults with Type 1 Diabetes: Analysis of the Type 1 Diabetes Exercise Initiative (T1DEXI) Cohort. Diabetologia.

[16] Morrison D., Vogrin S., Zaharieva D.P. (2024). Assessment of Glycemia Risk Index and Standard Continuous Glucose Monitoring Metrics in a Real-World Setting of Exercise in Adults with Type 1 Diabetes: A Post-Hoc Analysis of the Type 1 Diabetes and Exercise Initiative. J. Diabetes Sci. Technol..

[17] Codella R., Bisio A., Bassi M., Minuto N., Vichi E., Gotti D., Ruggeri P., Maggi D., Faelli E. (2025). Impact of Timing, Type, and Intensity of Physical Activity on Glycemic Outcomes in a Cohort of Well-Controlled Youth with Type 1 Diabetes. J. Endocrinol. Investig..

[18] Joubert M., Meyer L., Bekka S., Rakotoarisoa L., Scheyer N., Dreves B., Guerci B. (2025). Hypoglycemia Incidence and Behavioural Adjustments during Free-Living Unstructured Physical Activity in Adults with Type 1 Diabetes Using AID Systems: Results from the RAPPID Study. Diabetes Obes. Metab..

[19] Bergford S., Riddell M.C., Jacobs P.G., Li Z., Gal R.L., Clements M.A., Doyle F.J., Martin C.K., Patton S.R., Castle J.R. (2023). The Type 1 Diabetes and EXercise Initiative: Predicting Hypoglycemia Risk During Exercise for Participants with Type 1 Diabetes Using Repeated Measures Random Forest. Diabetes Technol. Ther..

[20] Ma S., Coopergard R., Clements M., Chow L. (2025). Managing Exercise-Related Glycemic Events in Type 1 Diabetes: Development and Validation of Predictive Models for a Practical Decision Support Tool. JMIR Diabetes.

[21] Jacobs P.G., Chase Marak M., Calhoun P., Gal R.L., Castle J.R., Riddell M.C. (2024). An Evaluation of How Exercise Position Statement Guidelines Are Being Used in the Real World in Type 1 Diabetes: Findings from the Type 1 Diabetes Exercise Initiative (T1DEXI). Diabetes Res. Clin. Pract..

[22] Moser O., Riddell M.C., Eckstein M.L., Adolfsson P., Rabasa-Lhoret R., Boom L., Gillard P., Nørgaard K., Oliver N.S., Zaharieva D.P. (2020). Glucose Management for Exercise Using Continuous Glucose Monitoring (CGM) and Intermittently Scanned CGM (isCGM) Systems in Type 1 Diabetes: Position Statement of the European Association for the Study of Diabetes (EASD) and of the International Society for Pediatric and Adolescent Diabetes (ISPAD) Endorsed by JDRF and Supported by the American Diabetes Association (ADA). Pediatr. Diabetes.

[23] McCarthy O.M., Christensen M.B., Kristensen K.B., Schmidt S., Ranjan A.G., Bain S.C., Bracken R.M., Nørgaard K. (2023). Automated Insulin Delivery Around Exercise in Adults with Type 1 Diabetes: A Pilot Randomized Controlled Study. Diabetes Technol. Ther..

[24] Phillip M., Nimri R., Bergenstal R.M., Barnard-Kelly K., Danne T., Hovorka R., Kovatchev B.P., Messer L.H., Parkin C.G., Ambler-Osborn L. (2023). Consensus Recommendations for the Use of Automated Insulin Delivery Technologies in Clinical Practice. Endocr. Rev..

[25] Dutta D., Kamrul-Hasan A.B.M., Jindal R., Mohindra R., Nagendra L., Bhattacharya S. (2025). Impact of Semaglutide Use on Glycemic and Metabolic Profile in Adults with Type 1 Diabetes Having Overweight or Obesity: A Systematic Review and Meta-Analysis. Medicine.

[26] Shah V.N., Akturk H.K., Kruger D., Ahmann A., Bhargava A., Bakoyannis G., Pyle L., Snell-Bergeon J.K. (2025). Semaglutide in Adults with Type 1 Diabetes and Obesity. NEJM Evid..

[27] Yardley J.E., Kenny G.P., Perkins B.A., Riddell M.C., Balaa N., Malcolm J., Boulay P., Khandwala F., Sigal R.J. (2013). Resistance Versus Aerobic Exercise: Acute Effects on Glycemia in Type 1 Diabetes. Diabetes Care.

[28] Yardley J.E., Kenny G.P., Perkins B.A., Riddell M.C., Malcolm J., Boulay P., Khandwala F., Sigal R.J. (2012). Effects of Performing Resistance Exercise Before Versus After Aerobic Exercise on Glycemia in Type 1 Diabetes. Diabetes Care.

[29] McCarthy O., Moser O., Eckstein M.L., Deere R., Bain S.C., Pitt J., Bracken R.M. (2019). Resistance Isn’t Futile: The Physiological Basis of the Health Effects of Resistance Exercise in Individuals with Type 1 Diabetes. Front. Endocrinol..

[30] Dial A.G., Monaco C.M.F., Grafham G.K., Romanova N., Simpson J.A., Tarnopolsky M.A., Perry C.G.R., Kalaitzoglou E., Hawke T.J. (2020). Muscle and Serum Myostatin Expression in Type 1 Diabetes. Physiol. Rep..

[31] Coleman S.K., Rebalka I.A., D’Souza D.M., Deodhare N., Desjardins E.M., Hawke T.J. (2016). Myostatin Inhibition Therapy for Insulin-Deficient Type 1 Diabetes. Sci. Rep..

[32] Bunn R.C., Adatorwovor R., Smith R.R., Ray P.D., Fields S.E., Keeble A.R., Fry C.S., Uppuganti S., Nyman J.S., Fowlkes J.L. (2023). Pharmacologic Inhibition of Myostatin with a Myostatin Antibody Improves the Skeletal Muscle and Bone Phenotype of Male Insulin-Deficient Diabetic Mice. J. Bone Miner. Res. Plus.

[33] Nunan E., Huff D.R., Gore J.L., Wright C.L., Harris T., Butler L., Padgett C.A., Rochowski M.T., Lovern P.C., Boolani A. (2025). Targeting Myostatin as an Adjunct Treatment for the Preservation of Cardiometabolic and Skeletal Muscle Function in Type 1 Diabetes. Int. J. Mol. Sci..

[34] Scott S., Kempf P., Bally L., Stettler C. (2019). Carbohydrate Intake in the Context of Exercise in People with Type 1 Diabetes. Nutrients.

[35] Muntis F.R., Mayer-Davis E.J., Shaikh S.R., Crandell J., Evenson K.R., Smith-Ryan A.E. (2023). Post-Exercise Protein Intake May Reduce Time in Hypoglycemia Following Moderate-Intensity Continuous Exercise among Adults with Type 1 Diabetes. Nutrients.

