# Supplementary material for: Glycemic Risk Across Exercise Modalities in Adults with Type 1 Diabetes Using Continuous Glucose Monitoring and Wearable Sensors: A Prospective Cohort Study
Source: J Funct Morphol Kinesiol. 2026 Jun 8;11(2):231. doi: 10.3390/jfmk11020231 (PMC13300763; doi:10.3390/jfmk11020231)
Supplement: Supplementary file 1 [file jfmk-11-00231-s001.zip › jfmk-4252494-supplementary.pdf]

Table S1. Practical nutrition considerations before, during, and after exercise in adults with type 1 diabetes.

Timing	Main goal	Practical consideration	Caution	Key supporting references
Before exercise	Reduce early glycemic instability and support safe exercise initiation	Individualize carbohydrate planning and insulin adjustment according to starting glucose, CGM trend, recent prandial insulin exposure, planned exercise modality, and expected session duration. If glucose is unexpectedly high or relative insulin deficiency is suspected, ketone assessment should be considered rather than reflex carbohydrate intake.	These considerations should not be interpreted as a standardized nutrition protocol. Reflex carbohydrate intake should be avoided when hyperglycemia may reflect insulin deficiency, and management should follow individualized clinical guidance.	[6,12,22,34]
During exercise	Limit excessive glucose decline, particularly during prolonged or predominantly aerobic activity	Use rapid-acting carbohydrate supplementation guided by CGM trends, session duration, exercise intensity, symptoms, and prior insulin exposure. Greater vigilance may be needed during aerobic sessions, which were associated with the highest rescue carbohydrate use in the present cohort.	Avoid overly prescriptive carbohydrate advice, because CGM lag, insulin on board, fitness level, exercise modality, and individual response can substantially modify glucose needs.	[6,10,22,34]
After exercise	Reduce delayed hypoglycemia and support recovery	Review post-exercise glucose trends, insulin dosing, and individualized carbohydrate replacement. Post-exercise protein intake may help lessen late glucose decline in some settings, but should be considered an emerging supportive strategy rather than a universal recommendation.	Evidence regarding protein timing and post-exercise hypoglycemia prevention is still emerging and should be interpreted cautiously. This should not be presented as a general supplement prescription.	[6,12,22,34,35]

Note: This table summarizes literature-informed practical considerations and was not derived from standardized nutrition or supplementation data collected in the present cohort. It should be interpreted as general clinical context rather than individualized dietary, supplementation, or insulin-management advice. CGM, continuous glucose monitoring.
